# Glucagon-Like Peptide-1 Receptor Ligand Interactions: Structural Cross Talk between Ligands and the Extracellular Domain

**DOI:** 10.1371/journal.pone.0105683

**Published:** 2014-09-02

**Authors:** Graham M. West, Francis S. Willard, Kyle W. Sloop, Aaron D. Showalter, Bruce D. Pascal, Patrick R. Griffin

**Affiliations:** 1 Department of Molecular Therapeutics, The Scripps Research Institute, Scripps Florida, Jupiter, Florida, United States of America; 2 Mass Spectrometry and Proteomics, The Scripps Research Institute, Scripps Florida, Jupiter, Florida, United States of America; 3 Informatics Core, The Scripps Research Institute, Scripps Florida, Jupiter, Florida, United States of America; 4 Quantitative Biology, Lilly Research Laboratories, Eli Lilly and Company, Indianapolis, Indiana, United States of America; 5 Endocrine Discovery, Lilly Research Laboratories, Eli Lilly and Company, Indianapolis, Indiana, United States of America; Van Andel Research Institute, United States of America

## Abstract

Activation of the glucagon-like peptide-1 receptor (GLP-1R) in pancreatic β-cells potentiates insulin production and is a current therapeutic target for the treatment of type 2 diabetes mellitus (T2DM). Like other class B G protein-coupled receptors (GPCRs), the GLP-1R contains an N-terminal extracellular ligand binding domain. N-terminal truncations on the peptide agonist generate antagonists capable of binding to the extracellular domain, but not capable of activating full length receptor. The main objective of this study was to use Hydrogen/deuterium exchange (HDX) to identify how the amide hydrogen bonding network of peptide ligands and the extracellular domain of GLP-1R (nGLP-1R) were altered by binding interactions and to then use this platform to validate direct binding events for putative GLP-1R small molecule ligands. The HDX studies presented here for two glucagon-like peptide-1 receptor (GLP-1R) peptide ligands indicates that the antagonist exendin-4_[9-39]_ is significantly destabilized in the presence of nonionic detergents as compared to the agonist exendin-4. Furthermore, HDX can detect stabilization of exendin-4 and exendin-4_[9-39]_ hydrogen bonding networks at the N-terminal helix [Val19 to Lys27] upon binding to the N-terminal extracellular domain of GLP-1R (nGLP-1R). In addition we show hydrogen bonding network stabilization on nGLP-1R in response to ligand binding, and validate direct binding events with the extracellular domain of the receptor for putative GLP-1R small molecule ligands.

## Introduction

Endogenous ligands of the glucagon-like peptide-1 receptor (GLP-1R) are small peptide hormones including glucagon-like peptide-1 (GLP-1), which is encoded by the glucagon gene [1] and these peptides are secreted from pancreatic beta-cells [2] The incretin hormones are potent stimulators of glucose-induced insulin biosynthesis and secretion. The plasma half-life of these peptide hormones is significantly limited by processing via the extracellular enzyme dipeptidyl peptidase-4 (DPP4). DPP4 resistant mimetics of these endogenous peptides such as exendin-4 and liraglutide, offer extended plasma half-life as compared to endogenous hormone and they are used therapeutically for the treatment of type 2 diabetes mellitus (T2DM) [3]. Small molecules targeting the GLP-1R would provide an attractive oral dosing alternative to the current peptide-derivative injectable therapeutics [4]. The glucagon-like peptide-1 receptor (GLP-1R) is a member of the secretin class (class B) G protein-coupled receptor (GPCR) family. Like other class B GPCRs, the GLP-1R consists of two main domains, a large extracellular ligand binding domain and a seven transmembrane helical domain [5], [6]. This extracellular domain is capable of binding the C-terminal side of peptide ligands, and is believed to orient the N-terminal region of peptide ligands toward the classic orthosteric transmembrane binding pocket [7], [8].

Crystal structures of the N-terminal extracellular domain of GLP-1R (nGLP-1R) show that the endogenous agonist, GLP-1, and the synthetic antagonist, exendin-4_[9-39]_, bind to the extracellular domain via interactions primarily between conserved side chains in the FxxWL motif of GLP-1R peptide ligands and Trp39 of nGLP-1R [9], [10]. The resolved regions of the peptide ligands adopt an α-helical secondary structure. The C-terminal amino acids of the peptide ligands are not resolved in the x-ray crystal structures, but solution-based NMR and circular dichroism (CD) studies [11], [12] indicate that the C-terminal side of exendin-4 adopts an α-helical secondary structure in aqueous phase and is unstructured in the presence of zwitterionic micelles consisting of dodecylphosphocholine (DPC). Questions remain regarding the secondary structure and regional stability of class B GPCR peptide ligands in the environment around a cell membrane. Recent advances in crystallography have used nonionic n-dodecyl-β-D-maltoside (DDM) and cholesterol hemisuccinate (CHS) and derivatives of these lipids to obtain near-full length GPCR structures in micelles, including two class B GPCRs, the human glucagon receptor (GCGR) and human corticotropin-releasing factor receptor 1 (CRF_1_) [13], [14]. These nonionic lipids are milder denaturants compared to their ionic counterparts and represent a more biologically relevant lipid membrane environment.

Potent antagonists can be generated from N-terminal truncations of agonist peptides. For example, the GLP-1R peptide agonist exendin-4 differs from the antagonist exendin-4_[9-39]_ by 8 amino acids at the N-terminus. While the functional effect of this truncation is well characterized, the structural consequences are not fully understood. In addition, many small molecule GLP-1R agonists have been reported recently [4]. Mass spectrometric-based quantitation of protein amide hydrogen exchange for deuterons from heavy water buffers can detect perturbations in a protein's conformational ensemble in response to ligand binding [15], [16]. In these experiments a bottom-up proteomic-based liquid chromatography/mass spectrometry (LCMS) platform is typically used to digest the deuterium labeled protein to facilitate determination of site-specific differential deuterium in-exchange. This technique has been applied to the study of GPCRs previously using mass spectrometry compatible detergents [17]. While this technique does not provide structural atomic resolution, it can detect differences in relative thermodynamic stability of hydrogen bonding networks at a resolution of a few amino acids. The spacial resolution of a bottom-up HDX experiment is limited by the generation and detection of peptides from pepsin proteolysis. Classic fragmentation techniques such as collision-induced dissociation (CID) impart excess vibrational energy to the peptides which promotes scrambling of deuterium label across all amides and side chains prior to fragmentation. Thus amide-specific information from the deuterium labeling of the protein is lost during scrambling. The low energy, radical-driven fragmentation technique, electron capture dissociation (ETD), has recently been shown to avoid excess vibration energy and reduce scrambling on model peptides [18]. This technique has been extended to the apo form of β_2_-microtubulin [19] and a nuclear receptor, peroxisome proliferator activated receptor, in the apo and ligand-bound state [20].

Here, we applied hydrogen/deuterium exchange (HDX) coupled to mass spectrometry to study the hydrogen bond network stability of exendin-4 and exendin-4_[9-39]_ in DDM/CHS micelles in the presence and absence of nGLP-1R at near physiological pH (7.5). Conformational changes at the FxxWL motif of peptide ligands were observed upon binding nGLP-1R. Fragmentation using ETD of a proteolytic peptide from exendin-4_[9-39]_ provided single amino acid resolution HDX at the binding site and indicated weaker thermodynamic stability at the hydrogen bond network in-between the two peptide ligand α-helices. In addition, we demonstrate that HDX-MS is capable of detecting conformational changes to the nGLP-1R upon binding peptide ligands and extend this to confirm binding interactions of proposed small molecule ligands.

## Experimental Procedures

### Reagents and Chemicals

Exendin-4 (cat # E7144), HPLC grade H_2_O, D_2_O (99.9%), acetonitrile, formic acid, tris-(carboxyethyl)-phosphinehydrochloride (TCEP), NaCl, NaH_2_PO_4_ and glycerol were purchased from Sigma-Aldrich. Exendin-4_[9-39]_ (cat # H-8740) and Oxyntomodulin (#H-6058) were purchased from Bachem. nGLP-1R (amino acids 24-145) was expressed and purified according to published methods [21]. Purified nGLP-1R was determined to be functional by radioligand binding. GLP-1(7-36) was provided by Eli Lilly. The GLP-1R ligands 6-BPPI (**6**-(2,5-dichloro**b**enzyl)-1-hydroxy-2-(2-morpholin-4-ylethyl)-1,6-dihydro**p**yrrolo[3′,4′:5,6]**p**yrido[3,4-B]**i**ndol-3(2H)-one) [22], Boc-5 [23], T-0632 (sodium (S)-3-[1-(2-fluorophenyl)-2,3-dihydro-3-[(3-isoquinolinyl-carbonyl)amino]-6-methoxy-2-oxo-1H-indole]propanoate) [24], [25] and TT15 (2S)-2-[[(8S)-7-benzoyl-3-[4-[(3,4-dichlorophenyl)methoxy]phenyl]-2-oxo-1,6,8,9-tetrahydropyrido[4,3-g][1,4]benzoxazine-8-carbonyl]amino]-3-[4-(4-cyanophenyl)phenyl]propanoic acid) [26] were synthesized according to standard or published methods at Eli Lilly. Trifluoroacetic acid (TFA, Sequanal grade) was obtained from Pierce. High purity n-dodecyl-β-D-maltoside (DDM) and cholesterol hemisuccinate (CHS) were purchased from Avanti Polar Lipids. The porcine pepsin-immobilized POROS 20 AL beads (particle size 20 µm) used to pack immobilized pepsin columns were purchased from Applied Biosystems. The model peptide for gas phase scrambling experiments, HHHHHHIIKIIK, was purchased from the American Peptide Company (#350416).

### Protein and Peptide Samples

All stock solutions and dilutions were made using the 7TM HDX buffer. The 7TM HDX buffers were composed of 50 mM Hepes (pH 7.5), 150 mM NaCl, 2% (v/v) glycerol, 0.05% (m/v) DDM, and 0.01% (m/v) CHS in either H_2_O or in D_2_O for on-exchange. All HDX stock solutions of the N-terminal extracellular domain of the glucagon-like peptide-1 receptor (nGLP-1R) were prepared at 10 µM nGLP-1R in the 7TM HDX H_2_O buffer. When ligands were present in the nGLP-1R stock solutions peptide ligands (exendin-4, exendin-4_[9-39]_, oxyntomodulin and GLP-1) were at 20 µM and small molecule ligands (6-BPPI, Boc5, T-0632 and TT15) were at 100 µM. All HDX stock solutions of the peptides were at 10 µM. When ligands were studied in the presence of the nGLP-1R the stock solutions contained 20 µM nGLP-1R. When ligands and receptor were mixed in stock solutions they were allowed to equilibrate for one hour at 4°C.

### Hydrogen/Deuterium Exchange

Dilution of 5 µL of stock solutions into 20 µL of D_2_O buffers initiated on-exchange and resulted in an 80% D_2_O environment. Solution-phase amide HDX was carried out with a fully automated system described previously [17]. Briefly, the solution handling and mixing was performed with a LEAP Technologies Twin HTS PAL liquid handling robot and a temperature controlled cabinet held at 4°C. On-exchange was carried out for predetermined times (10, 30, 60, 900 and 3600 seconds) at 4°C before adding all 25 µL of the exchange reaction to 25 µL of quench solution. The quench solution contained 100 mM NaH_2_PO_4_, 0.02% DDM, and 15 mM TCEP at pH 2.4. Digestion was performed in line with chromatography using and in-house packed pepsin column at a flow rate of 50 µL/min. Peptides were captured and desalted on a 2 mm i.d. C8 trap (Thermo Fisher Scientific). Peptides were then separated across a 5 µ 10×1 mm Betasil C8 column (Thermo Fisher Scientific) with a linear gradient of 12–40% acetonitrile in 0.3% formic acid over a short five minute gradient to limit back exchange with the solvent. Three to four replicates were performed for each HDX time point.

Mass spectra were acquired in the range of m/z 300–2000 at a resolution of 60,000 for 8 min in positive ion mode on an LTQ Orbitrap XL ETD mass spectrometer (Thermo Fisher Scientific) equipped with an ESI source operated at capillary temperature of 225°C and spray voltage of 3.5 kV. The intensity weighted average m/z value (centroid) of each peptide's isotopic envelope was calculated with in-house developed software; Workbench (Pascal et al. 2013) and converted to % Deuterium values. The corrections for back-exchange for exendin-4 and exendin-4_[9-39]_ were determined experimentally for each peptic peptide by performing an off-line digest of each sample and carrying out one hour on-exchange for the digested mixture using the same methods that were used for samples. For all other samples back-exchange correction was based on an estimated 70% deuterium recovery and accounting for the known 80% deuterium content of the on-exchange buffer. The Workbench software used P-values lower than 0.05 for two consecutive time points were used to determine significance.

### MS/MS Sequencing

Sequence coverage experiments for these proteins were carried out in the 7TM HDX buffer and LC system described above, but with a longer 60 minute gradient. 50 pmols of nGLP-1R or peptide were loaded on column. For sequencing tandem mass spectra were obtained using data-dependent acquisition with 30 second dynamic exclusion where the top five most abundant ions in each scan were selected and subjected to CID fragmentation. Each scan was the average of 3 microscans under normal scan mode in both MS and MS/MS.

### Controls for Electron Transfer Dissociation

To optimize the instrument parameters and to monitor gas-phase scrambling, we employed the model peptide HHHHHHIIKIIK described by the Jorgensen group. Complete deuteration was achieved by incubation of the peptide in deuterated buffer (20 mM NaH_2_PO_4_, pH 2.6) for one hour at 4°C. HDX off-exchange was initiated by a 10-fold dilution into infusion buffer (50% MeOH, 0.5 M CH_3_COOH, pH 2.6) followed by elution over the same LC configuration used for HDX after 30 s of off-exchange. Under these conditions the peptide eluted in ∼1.6 minutes. Full-scan and ETD MS/MS spectra were acquired on the LTQ Orbitrap XL ETD. Instrument conditions were identical to those published previously except the capillary temperature was set to 75°C. Varying isolation widths and capillary temperatures were tested to minimize scrambling and maintain ion signal intensity (Figure S3). A capillary temperature of 75°C and isolation width of 12 amu was used for ETD of exendin-4_[9-39]_. Five replicates were run after 30 s of deuterium exchange for exendin-4_[9-39]_ in the presence and absence of nGLP-1R on the same LC configuration described above.

### Cellular Assays

cAMP accumulation assays were performed as previously described [27]. HEK293 cells were transfected with plasmids expressing either wild-type GLP-1R (NP_002053) or chimera nGIP-R:GLP-1R (amino acids 1-124 of GIP-R (NP_000155) fused in-frame with amino acids 137-463 of GLP-1R).

## Results

**Figure 1:**
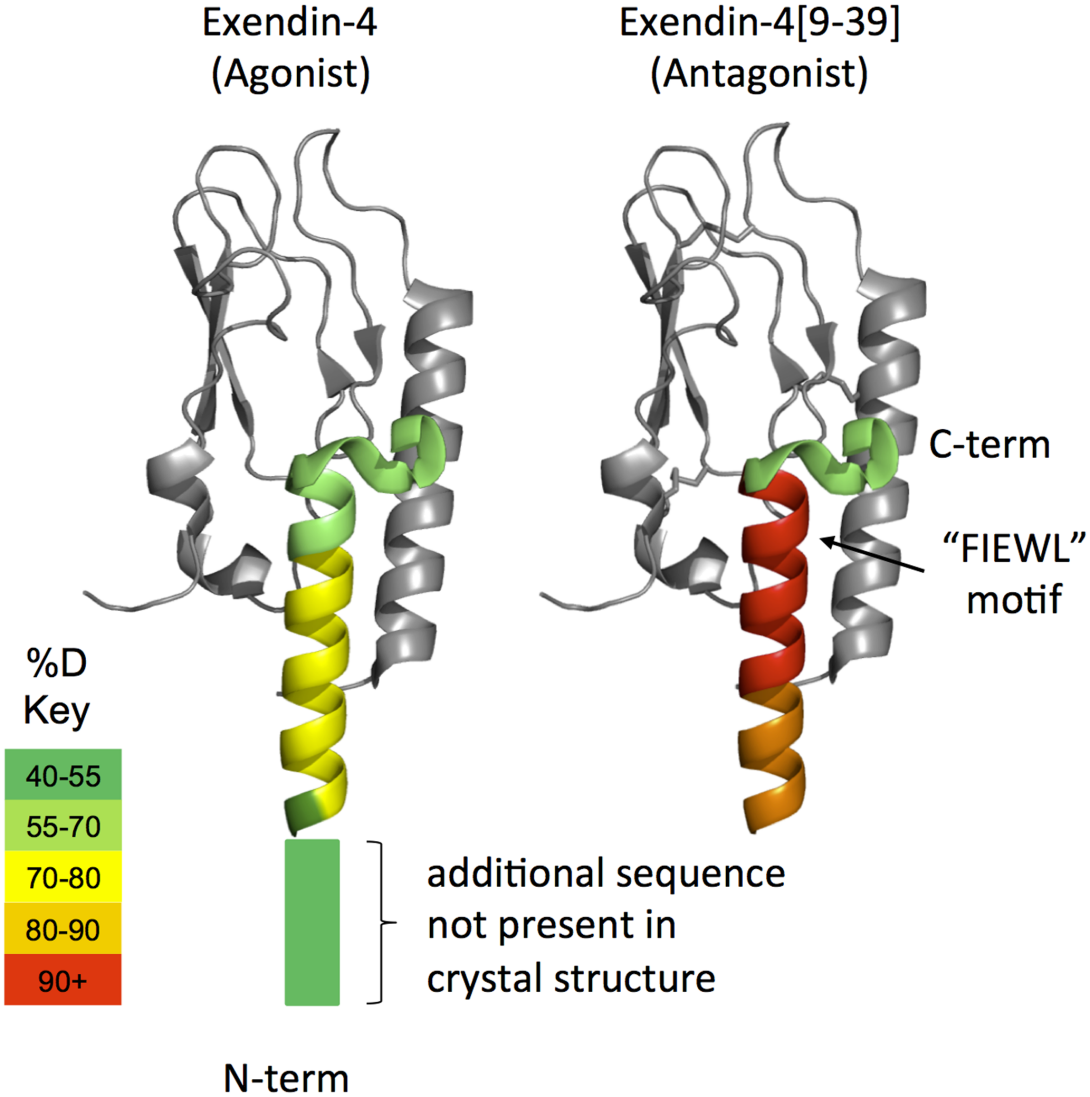
HDX of Peptide Ligands Color Coded onto Crystal Structure. Average % deuterium exchange values for exendin-4 and exendin-4_[9-39]_ overlaid onto the crystal structure pdb:3C5T. Deuterium exchange was measured in the presence of DDM/CHS micelles as indicated in the Experimental section. The nGLP-1R was not present in these samples, but is included in gray for visual reference. As shown in the key, color indicates the average % deuterium uptake across all five time points (10 s to 1 hr) for digested peptic peptides within each region of the peptide ligands. Deuterium exchange at additional residues specific to the agonist (not present in the antagonist crystal structure shown) are indicated with the rectangular box on the N-terminal side of the ligand.

Differential HDX [15] was measured for the agonist, exendin-4, and an N-terminal truncated version, exendin-4_[9-39]_, a potent antagonist, in the presence of DDM/CHS micelles in an aqueous solution to mimic the environment near a cell surface. Full deuterium in-exchange was determined experimentally and used to correct peptide-specific and retention time specific deuterium uptake variations (see methods). Lower deuterium incorporation was observed for all homologous peptides of the full length exendin-4 (Figure 1 and Figure S1). Lower deuterium incorporation was also observed for non-homologous peptides as a result of inefficient pepsin cleavage at S8 on exendin-4 (Figure S1).

**Figure 2:**
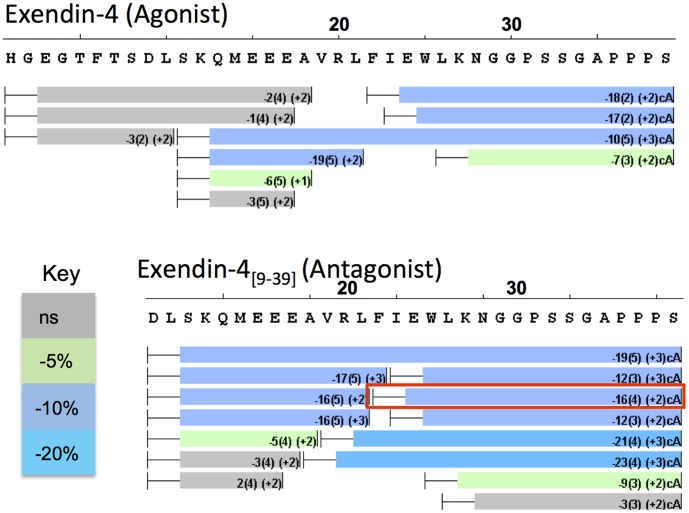
Stabilization of Peptide Ligands by nGLP-1R. A ‘perturbation map’ showing changes to deuterium exchange for peptide ligands in the presence and absence of nGLP-1R. Deuterium exchange was measured in the presence of DDM/CHS micelles as indicated in the Experimental section. Resolved peptides are indicated with rectangular boxes below the sequence of exendin-4 and exendin-4_[9-39]_. The whiskers on the left side of each bar indicate amino acids that are included in the detected peptides, but whose amides do not report on deuterium in-exchange. As a result of digestion the first amino acid no longer contains an exchangeable amide and the back exchange rates are higher for the second amino acid. The average % change in deuterium in the presence of nGLP-1R is included inside the boxes with standard error in parenthesis. Boxes are colored according to the key where significant changes are determined by t-test. The peptide enclosed in the red box was chosen for ETD fragmentation.

The extracellular domain of GLP-1R is known to bind exendin-4 and exendin-4_[9-39]_ with high affinity [21]. In order to compare stabilization of these ligands by the receptor extracellular domain HDX was performed on the peptide ligands in the presence of nGLP-1R (Figure 2). Given the timescale and automation inherent in our HDXMS approach we performed all analyses in triplicate to enable the use of statistical analyses (i.e. t-tests) to determine if changes observed between states were significant. When HDX was measured for these peptides bound to nGLP-1R, protection to exchange was observed at the N-terminal helix near the bend, the same region in close contact with nGLP-1R in crystal structures [9], [10]. However, the N-terminal amino acids His1 to Glu17 showed no statistically significant change in the magnitude of deuterium exchange, and as such showed no evidence for stabilization at this region of the peptide upon binding nGLP-1R (Figure 2). No significant protection was observed for the endogenous agonist, GLP-1 (Figure S2).

**Figure 3:**
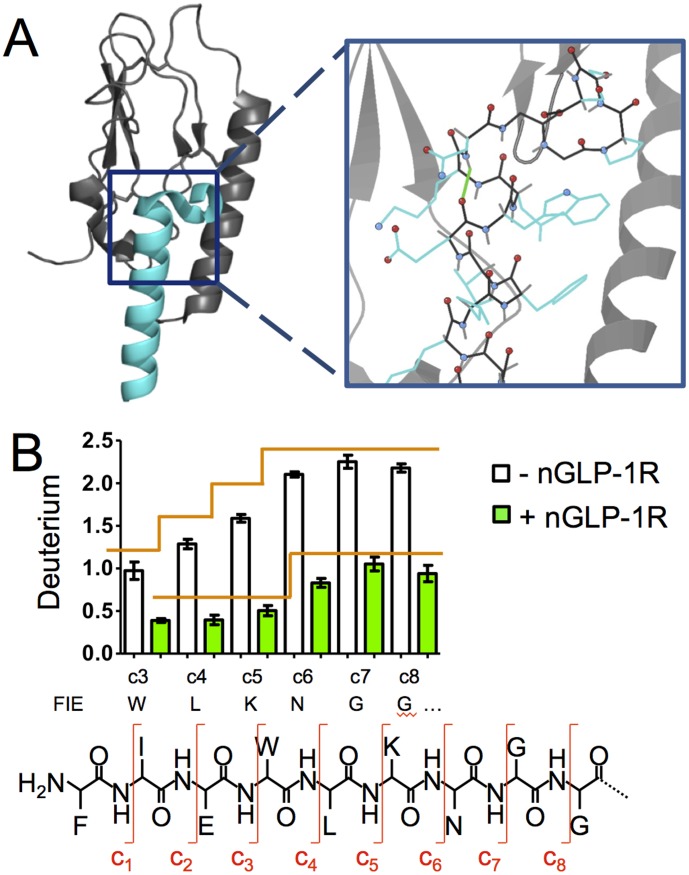
HDX at Single Amino Acid Resolution for Exendin-4_[9-39]_. A) Top: Crystal structure of exendin-4_[9-39]_ (teal) bound to nGLP-1R (Gray), pdb3C5T. Bottom: The location of the backbone amide hydrogen bond on Asn28 is shown in green in the zoomed in image. B) Deuterium exchange on individual amino acids from ETD data for exendin-4_[9-39]_ in the absence and presence of nGLP-1R. Deuterium exchange was measured in the presence of DDM/CHS micelles as indicated in the Experimental section. The average number of deuterium atoms exchanged is shown for each c fragment ion with the corresponding amino acid. Note that the c6 amide contains the Lys27 side chain, but the amide is associated with Asn28 in the sequence. A detailed structure of the peptide and c fragments is shown below the ETD data for reference. Significant increases in deuterium exchange as determined by Tukey analysis are indicated with vertical transitions on the orange lines. Each deuterium exchange measurement is the average of 5 replicates after 30 s of exchange.

In a bottom-up HDX experiment the deuterium exchange in digested peptides reports on the exchange from the intact protein, as digestion occurs after the exchange reaction has been quenched. The spacial resolution of a bottom-up HDX experiment is limited by the length of the proteolytic peptides generated upon digestion, where deuterium exchange cannot be calculated for specific amides, but rather for the sum of all of the amides within a given peptide. Despite the high degree of overlapping peptides, it is difficult to accurately calculate single amino acid amide exchange using subtractive analysis. Many HDX studies that report subtractive analysis for single amino acid resolution overlook error propagation and back exchange correction, which makes determination of statistical significance difficult if not impossible [16]. For this reason a soft peptide ion fragmentation technique, electron transfer dissociation (ETD), was used to follow deuterium exchange at specific peptide fragments and thus achieve single amino acid resolution HDX [18]–[20], [28]. The exendin-4_[9-39]_ Phe22-Ser39 peptic peptide was chosen for ETD fragmentation based on high abundance for the intact triply charged ion and overlap with the turn region in the crystal structure [9] (Figure 2 and Figure 3). ETD settings were optimized to minimize deuterium scrambling (Figure S3). After 30 s of deuterium exchange, fragment ions corresponding to the amide hydrogens located on the C-terminal side of Glu24 to Gly29 were reproducibly resolved for each of five replicates in the apo and nGLP-1R bound state. Single amino acid resolution with ETD showed increasing deuterium incorporation for fragments from Trp25 to Asn28, signifying a weaker H-bond network in the absence of nGLP-1R (Figure 3). This deuterium incorporation plateaus after Asn28, the hydrogen bond at the turn at the end of the α-helix in the crystal structure [9], which supports formation of a C-terminal helix in the apo state. Significant protection is imparted by nGLP-1R at Trp25, Leu26 and Lys27, which supports stabilization of secondary structure upon receptor binding. The ETD fragmentation technique generated a series of “c” fragments which cleaves the peptide at the bonds shown in the diagram of Figure 3B. A small but statistically significant increase in deuterium incorporation was seen at the Asn28 turn in the presence of nGLP-1R.

**Figure 4:**
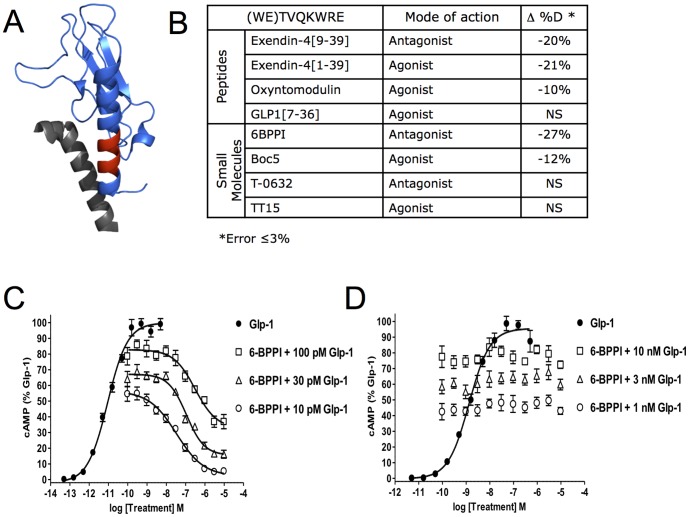
Changes to HDX of nGLP-1R in the Presence of Peptide and Small Molecule Ligands. A) The WETVQKWRE peptide of nGLP-1R where protection was observed is shown in red on the crystal structure pdb:3C5T. Deuterium exchange was measured in the presence of DDM/CHS micelles as indicated in the Experimental section The exendin-4_[9-39]_ peptide ligand is colored gray. B) Table showing the average change in % Deuterium in this same nGLP-1R peptide (WETVQKWRE) for four peptide ligands and four proposed small molecule ligands. GLP-1 induced cAMP accumulation was quantified in HEK293 cells transfected with plasmids expressing either (C) the GLP-1R or (D) a GIPR/GLP-1R chimeric protein where the GLP-1R 7TM domains are fused in-frame with the GIP-R ectodomain. Dose response curves of GLP-1 were generated and robust cAMP accumulation was observed for the GLP-1R (EC_50_ = 9.0 pM) and GIPR/GLP-1R (1.3 nM). The ability of 6-BPPI to antagonize GLP-1 signaling was measured by treatment of cells with a dose-response of 6-BPPI in the presence of EC_50_ to EC_80_ concentrations of GLP-1. Dose-dependent blockade of GLP-1R signalling by 6-BPPI (100 pM GLP-1, IC_50_ = 310 nM; 30 pM GLP-1, IC_50_ = 110 nM, 10 pM GLP-1, IC_50_ = 33 nM) but not GIPR/GLP-1R was observed.

When HDX was monitored for nGLP-1R in the presence and absence of exendin-4 and exendin-4_[9-39]_ only one region of the receptor sequence showed differences in deuterium incorporation (Figure 4). Peptides spanning Trp32 to Glu40 showed significant protection to exchange (Figure S4A). This was not surprising, as this region of nGLP-1R makes close contacts with peptides in crystal structures. Absence of protection at nGLP-1R loops that also make ligand contacts in the crystal structure [9] could result from weak stabilization of amide hydrogen bonds beyond the sensitivity of this HDX method or from the stabilizing interactions that are primarily mediated through side chain interactions. Interactions with two additional peptide agonists were tested to assess the sensitivity of nGLP-1R HDX (Figure 4B). Although protection to exchange was observed in the presence of oxyntomodulin, no significant change to deuterium exchange was observed for GLP-1.

Small molecule ligands represent attractive therapeutics to advance T2DM pharmacology [4], [27]. HDX of nGLP-1R was used as a platform to validate interactions with four small molecules whose scaffolds represent potential T2DM therapeutics or potential antagonists. HDX could confirm thermodynamic stabilizing interactions for two of the small molecules tested, 6-BPPI [22] and Boc-5 [23] (Figure 4B). The HDX data suggest that 6-BPPI interacts with nGLP-1R. To demonstrate this with an orthogonal method, we measured the effect of 6-BPPI on GLP-1 induced cAMP accumulation in GLP-1R transfected cells (Figure S4B and Figure S4C). 6-BPPI was an effective antagonist of the GLP-1R but was inactive on a chimeric receptor composed of the ectodomain of the gastric inhibitory polypeptide receptor (GIPR) fused to the 7TM domains of the GLP-1R.

## Discussion

Lower deuterium incorporation for full length exendin-4 (Figures 1 and S1) compared to the antagonist indicates that deletion of the eight N-terminal amino acids of the agonist peptide destabilizes secondary structure in solution. Based on the evidence of α-helical formation from X-ray [9], NMR [11] and CD [12] studies, these data suggest that agonist peptides sample an alpha-helical conformation more frequently than their antagonist counterparts. These experiments were carried out in the presence of detergent to mimic the biphasic hydrophobic environment around the surface of a cell membrane. Although these detergents are not necessary for solubility of the nGLP-1R or the ligands, they are necessary for full length GPCRs [17] and should make comparisons with future studies on full length receptor easier.

In contrast to the NMR data for exendin-4 in DPC [11], our study suggests that the C-terminal Trp-cage of exendin-4 is structured in solution in the presence of DDM/CHS micelles. This may be due to differences in micelle composition. DDM/CHS micelles were chosen for our study to complement current crystallography efforts and to more closely resemble a mammalian cell membrane environment. An energetically favorable insertion of the tryptophan ring into the DPC micelle was previously suggested as the basis for destabilization of the Trp-cage. We propose that insertion of the tryptophan ring may be detergent/micelle dependent. In a biological system, affinity of GLP-1R ligands for the cell membrane would help to recruit ligands to the vicinity of the receptor. However, dissociation from the membrane may be necessary for receptor-binding, so interactions at the membrane/solution interface could be critical for ligand recognition.

Amide hydrogens in exendin-4 and exendin-4_[9-39]_ showed protection to their hydrogen bonding network around the FxxWL motif (Figure 2). Overlapping sequences of peptic peptides confine HDX protection from Ala18 to Asn28. However, no change in deuterium exchange was observed at the N-terminal amino acids preceding Glu17, and as such there was no evidence for stabilization or destabilization at this region of the peptide upon binding nGLP-1R. It is likely that the N-terminal amino acids in peptide ligands, or at least agonists, make additional interactions with the classic orthosteric GLP-1R binding pocket or with the TM1 stalk region as proposed in the GCGR structure [13]. The data here suggests that the extracellular domain of GLP-1R may only help to orient peptide ligands in the two step binding model [5], [8] and does not impart any additional structural stability to the N-terminal amino acids of these ligands. No protection to exchange was observed for GLP-1, presumably due to the weaker affinity for the extracellular domain [21].

The spacial resolution of protection was limited using peptide subtractive analysis (see Results). ETD was employed to obtain single amino acid resolution HDX with lower standard deviations than found using a subtractive analysis approach. Single amino acid HDX using ETD indicates that increased thermodynamic stability is imparted to Trp25, Leu26 and Lys27 by binding to nGLP-1R, which implies a shift in conformational equilibrium favoring secondary structure. In the presence of nGLP-1R the amide hydrogen at Asn28 acquires 0.33 deuterium after 30 s of exchange (Figure 3). This deuterium exchange is in contrast to surrounding amides, which exchange very little if any deuterium after 30 s, and is evidence of a thermodynamically weaker hydrogen bond at this amide. This is the last amide hydrogen bond before the turn. Although this amide hydrogen appears to make a hydrogen bond within the alpha-helix in the nGLP-1R-bound crystal structure, the adjacent side chains N-terminal of this H-bond, Glu24 and Lys27, make ionic interactions with the receptor [9]. In addition to strain imparted by the helix turn, these interactions with the receptor could place further strain on this hydrogen bond and offset any thermodynamic stabilization afforded by secondary structure. It is noteworthy that ETD with HDX is able to pick up on subtle changes to deuterium exchange in solution with single amide resolution. At this time, single amino acid HDX with ETD is relatively new in the HDX field. The power of this technique is expected to be of great interest to HDX users, however instrument costs, a requirement for high intensity triply charged ions and a lack of supporting software likely limit its use. Extending current HDX data software [29]–[35] capabilities for intact peptides to include ETD fragment ions is expected to increase the use of ETD applications in this field.

Although protection to exchange was observed for nGLP-1R in the presence of exendin-4, exendin-4_[9-39]_ and oxyntomodulin (Figure 4B) no significant change to deuterium exchange was observed for GLP-1. This is presumably due to affinities of these respective ligands for nGLP-1R [36]. This highlights that structurally distinct GLP-1R peptide ligands can exhibit unique HDX signatures, and therefore HDX analysis may be a useful approach for comparing binding mechanisms of peptide ligands. Structures for the apo- and ligand-bound state of two other class B GPCR extracellular domains, the corticotrophin release factor receptor (CRFR) [37] and the type 1 parathyroid hormone receptor (PTH1R) [38], did not show evidence of conformational change at the N-terminal α-helix. However, the HDX data here show a significant thermodynamic stabilization at this region, indicating a conformation shift favoring formation of secondary structure. Interestingly, ligand binding in the CRFR structures indicates a slight (5–7 A) conformational change at the loop connecting the β3 and β4 strands. However, HDX data did not detect a thermodynamic stabilization at the hydrogen bond network in this region. This may indicate that ligand binding interactions here are primarily mediated through side chain interactions, or that any stabilization to amide hydrogen is below the limit of detection with this approach.

Given the HDX technique's success detecting GLP-1R peptide ligand interactions we decided to explore its use as a robust approach for ligand screening to validate the ability of proposed small molecule ligands to bind the extracellular domain of GLP-1R. HDX has been used as a robust approach for ligand screening previously with VDR [39]. Here, we tested this HDX nGLP-1R screening ability on four additional ligands, all considered ‘small molecule’ ligands, as a proof of principal study. In addition, some questions remain regarding binding site location for small molecule ligands. In theory small molecule ligands could bind to the extracellular domain, the classic transmembrane orthosteric pocket or both. The HDX experiments here are capable of confirming interactions with the extracellular domain. Protection to exchange was seen for two of the four ligands, 6-BPPI [22] and Boc-5 [23] (Figure 4B). It is possible that the other two ligands have weaker affinity for the extracellular domain, like GLP-1 [21], or interact with the classic orthosteric pocket. Larger non-peptide agonists such as Boc5 may be able to interact with both the extracellular domain and orthosteric pocket and may have higher affinity for full length GLP-1R. The HDX protection to the extracellular receptor domain here indicates binding interactions for both of these ligands occur at the extracellular domain of GLP-1R. This was confirmed for 6-BPPI using a cAMP assay on chimeric receptors in an HEK cell line (Figure S4B and S4C). These cellular assays confirm that 6-BPPI binds the extracellular domain and not the classic orthosteric transmembrane pocket. The observed protection for five of the ligands tested in Figure 4B demonstrates HDX of nGLP-1R can be implemented as a method to validate interactions with nGLP-1R for both peptides and small molecules.

A more detailed characterization of peptide interactions with membrane proteins is expected to be of interest as ∼30% of all cellular proteins are estimated to be integral membrane proteins [40] many of which hold therapeutic potential [41]. Future studies on the full length receptor and chimeric receptors are expected to provide additional information on ligands that either interact with the orthosteric transmembrane activation site or have reduced affinity for the extracellular domain like GLP-1. In addition, crystallography of GPCRs has seen recent breakthroughs and an additional understanding of GPCR peptide ligand behavior in the presence of detergents used to obtain these crystal structures is of high biological importance. Additional HDX studies on full length class B receptor constructs should extend structural signaling models and provide further characterization of this unique class of GPCRs and their ligands.

## Supporting Information

Figure S1 **HDX of Exendin-4 and Exendin-4_[9-39]_.** Proteolytic peptides from pepsin digest and deuterium in-exchange shown for exendin-4 and exendin-4_[9-39]_. Peptic peptides are represented using rectangular bars below the ligand sequence. The first number within each peptide bar indicates the average % deuterium measured for all time points after correction for back exchange. The first parenthetical number is the standard deviation associated with the measurement and the second is the peptide ion charge. The Key shows colors assigned to % deuterium ranges.
(TIFF)

Figure S2 **HDX for GLP-1.** A ‘perturbation map’ showing rectangular boxes where peptides were detected in the HDX experiment below the sequence of GLP-1. The average % change in deuterium in the presence of nGLP-1R is included inside the boxes with standard error in parenthesis. No changes in deuterium exchange were determined to be significant by t-test. See Supplemental Figure 1 for a more detailed description.
(TIFF)

Figure S3 **ETD Control Experiments to Minimize Scrambling.** Isotopic distributions of the c6 and z7, singly charged fragments from the +3 precursor ion of the HHHHHHIIKIIK peptide. Isotopic distributions are shown after no deuterium labeling, after deuterium labeling under conditions that minimize deuterium scrambling with a heated capillary temperature of 75°C and isolation window width of 12 amu, and after deuterium labeling with conditions that promote scrambling with a heated capillary temperature of 225°C and isolation window width of 3 amu.
(TIFF)

Figure S4 **Changes to HDX of nGLP-1R by Exendin-4 & Cellular Assays Confirming 6-BPPI antagonism of GLP-1R is Ectodomain Dependent.** A) A ‘perturbation map’ showing rectangular boxes where peptides were detected in the HDX experiment below the sequence of nGLP-1R. The average % change in deuterium in the presence of the agonist exendin-4 is included inside the boxes with standard error in parenthesis. Boxes are colored according to the key where changes are significant as determined by t-test.
(TIFF)
