# Supplementary figures and images for: Glucagon-Like Peptide-1 Receptor Ligand Interactions: Structural Cross Talk between Ligands and the Extracellular Domain
Source: PLoS One. 2014 Sep 2;9(9):e105683. doi: 10.1371/journal.pone.0105683 (PMC4152014; doi:10.1371/journal.pone.0105683)

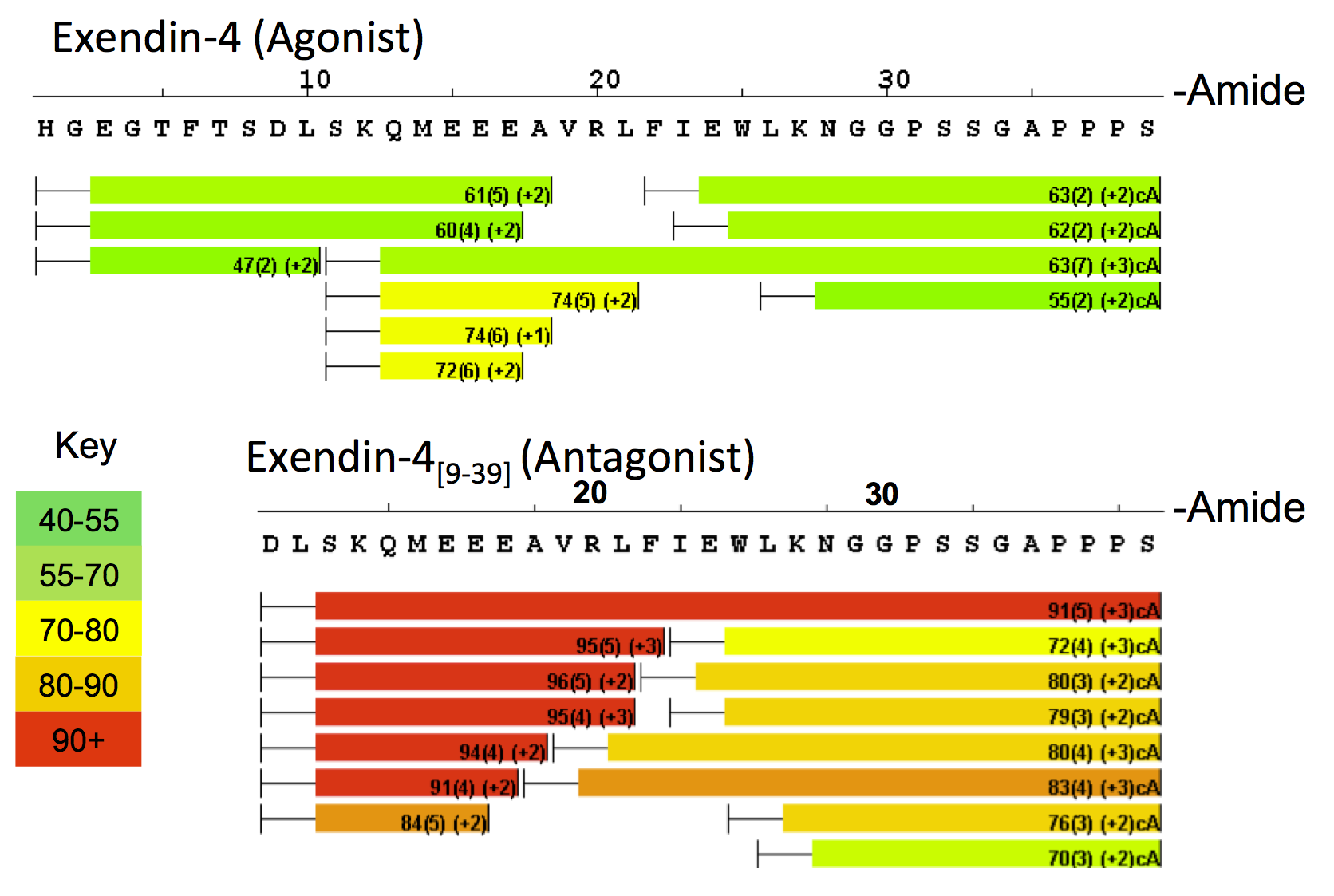

Supplement: Figure S1 — HDX of Exendin-4 and Exendin-4[9-39]. Proteolytic peptides from pepsin digest and deuterium in-exchange shown for exendin-4 and exendin-4[9-39]. Peptic peptides are represented using rectangular bars below the ligand sequence. The first number within each peptide bar indicates the average % deuterium measured for all time points after correction for back exchange. The first parenthetical number is the standard deviation associated with the measurement and the second is the peptide ion charge. The Key shows colors assigned to % deuterium ranges. (TIFF) [file pone.0105683.s001.tiff]

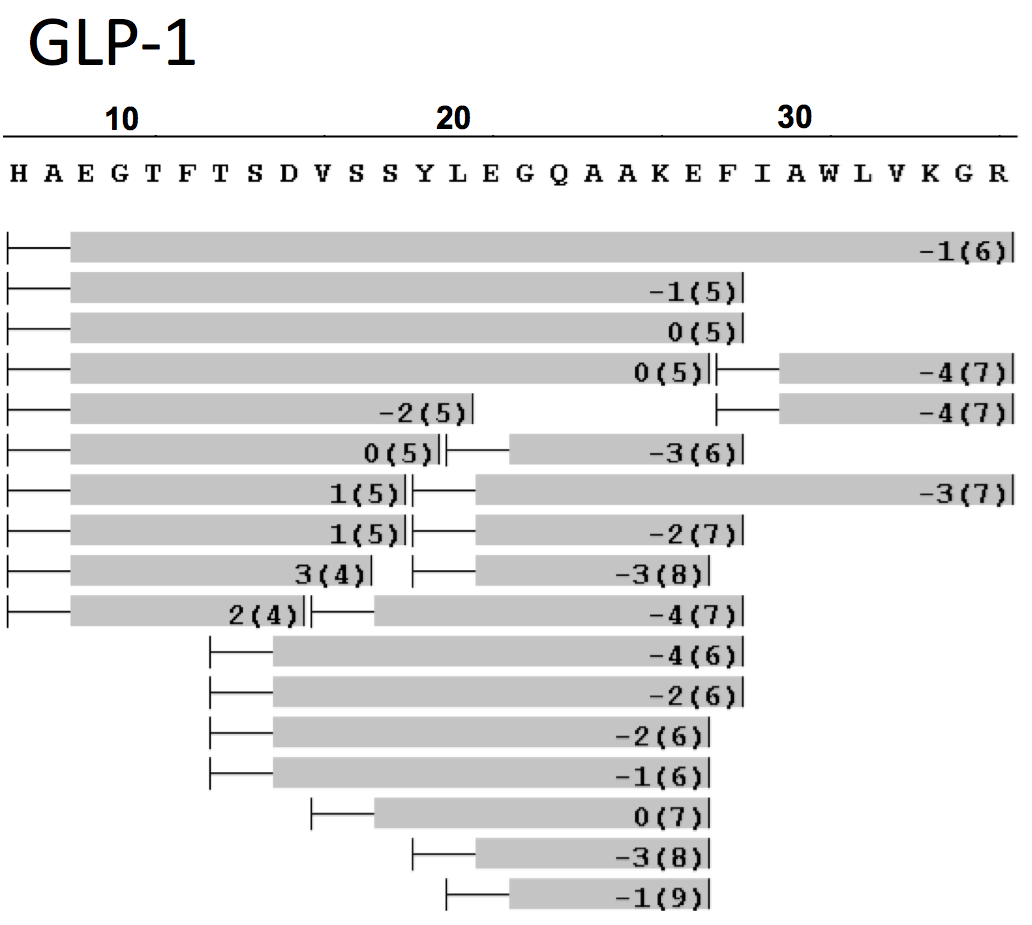

Supplement: Figure S2 — HDX for GLP-1. A ‘perturbation map’ showing rectangular boxes where peptides were detected in the HDX experiment below the sequence of GLP-1. The average % change in deuterium in the presence of nGLP-1R is included inside the boxes with standard error in parenthesis. No changes in deuterium exchange were determined to be significant by t-test. See Supplemental Figure 1 for a more detailed description. (TIFF) [file pone.0105683.s002.tiff]

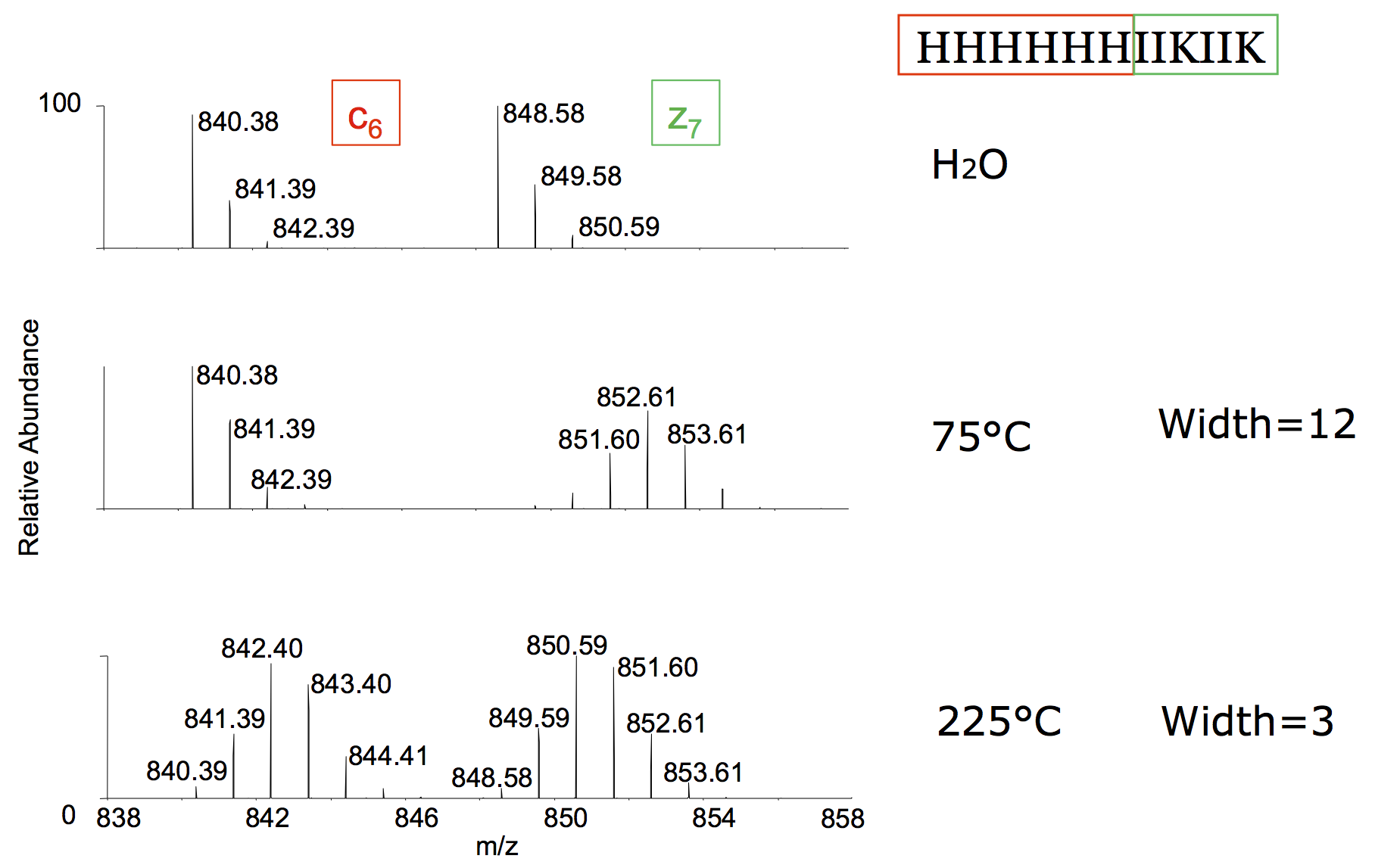

Supplement: Figure S3 — ETD Control Experiments to Minimize Scrambling. Isotopic distributions of the c6 and z7, singly charged fragments from the +3 precursor ion of the HHHHHHIIKIIK peptide. Isotopic distributions are shown after no deuterium labeling, after deuterium labeling under conditions that minimize deuterium scrambling with a heated capillary temperature of 75°C and isolation window width of 12 amu, and after deuterium labeling with conditions that promote scrambling with a heated capillary temperature of 225°C and isolation window width of 3 amu. (TIFF) [file pone.0105683.s003.tiff]

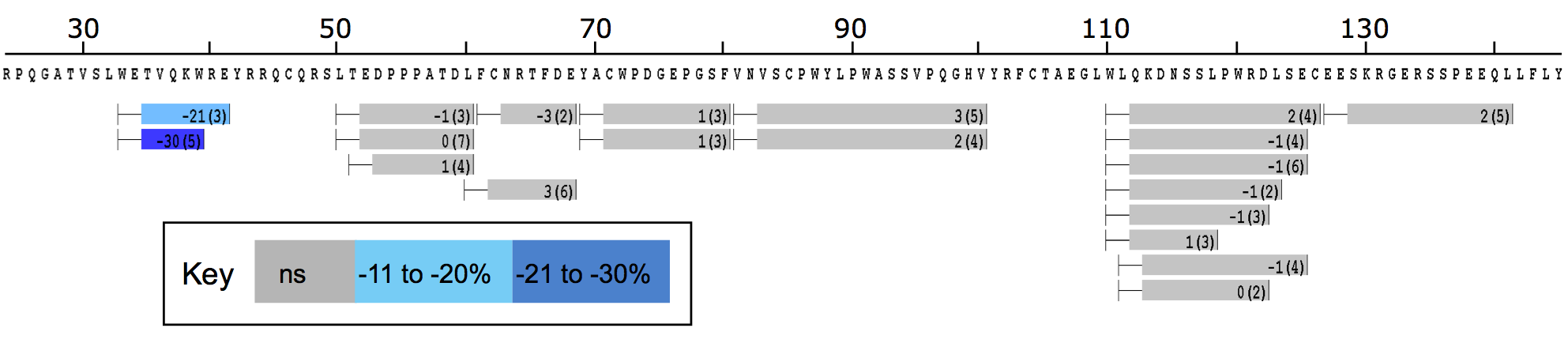

Supplement: Figure S4 — Changes to HDX of nGLP-1R by Exendin-4 & Cellular Assays Confirming 6-BPPI antagonism of GLP-1R is Ectodomain Dependent. A) A ‘perturbation map’ showing rectangular boxes where peptides were detected in the HDX experiment below the sequence of nGLP-1R. The average % change in deuterium in the presence of the agonist exendin-4 is included inside the boxes with standard error in parenthesis. Boxes are colored according to the key where changes are significant as determined by t-test. (TIFF) [file pone.0105683.s004.tiff]
